# Simulation dataset on power and size of Wald, likelihood-ratio, and score test statistics in parametric competing risks models under hybrid censoring

**DOI:** 10.1016/j.dib.2026.113136

**Published:** 2026-08-04

**Authors:** Nargiza S. Nurmukhamedova

**Affiliations:** National University of Uzbekistan named after Mirzo Ulugbek, University str. 4, Tashkent, 100174, Uzbekistan

**Keywords:** Competing risks, Hybrid censoring, Monte Carlo simulation, Maximum likelihood estimation, Wald statistic, Likelihood-ratio test, Power, Fisher information

## Abstract

This data article describes a simulation dataset generated for parametric competing risks models (CRM) with *K* = 2 or *K* = 3 independent competing causes of failure under hybrid (Type I/II combined) censoring — a scheme that combines a time limit $T_{0}$ (Type I) and a failure-count limit r (Type II), common in accelerated life testing and clinical trials. The dataset was produced by Monte Carlo simulation in Python 3.11 (*R* = 5000 replications; base seed 42; independent parallel streams). Three parametric families are included: exponential, Weibull (α = 2), and Gompertz. The experimental grid covers *n* ∈ {50, 100, 200, 500} and β ∈ {0.25, 0.50, 0.75, 1.00}. For each combination the dataset records: (i) empirical bias and RMSE of the MLE together with the theoretical prediction from the Fisher information identity $I\left(\theta_{0}\right)=\beta I^{M}\left(\theta_{0}\right)$; (ii) empirical Type I error and power of the Wald ($W_{n}$), likelihood-ratio ($LR_{n}$), and score ($S_{n}$) statistics at α = 0.05; (iii) non-centrality parameter λ* and theoretical power; (iv) asymptotic and bootstrap 95 % CI coverage at *n* ∈ {30, 50, 100}; (v) Type I error under Clayton copula dependence; (vi) required minimum sample size n*. A diagnostics file and an application to the survival::lung dataset (*n* = 228) are also included. All 16 files are deposited in Mendeley Data (DOI: 10.17632/mv42gshrcv.2). This dataset enables researchers to: (i) benchmark new statistical methods for competing risks under hybrid censoring without rerunning all simulations; (ii) empirically validate theoretical Fisher information predictions from $I\left(\theta_{0}\right)=\beta I^{M}\left(\theta_{0}\right)$; (iii) plan sample sizes for clinical trials and reliability tests using the n* tables; and (iv) reproduce or extend the simulation framework via the provided Python and R scripts.

Specifications Table

**Table:** 

Subject	Mathematics and Statistics
Specific subject area	Parametric competing risks models; maximum likelihood estimation; hypothesis testing; power analysis; hybrid censoring; Monte Carlo simulation
Type of data	Table (.csv) — Processed, Analyzed Script (.py, .R)
Data collection	Monte Carlo simulation: Python 3.11 (NumPy 1.24, SciPy 1.11). R = 5000 replications per (n, β, model) setting. Independent SeedSequence child streams per parallel worker (base seed 42). *n* ∈ {50, 100, 200, 500}; β ∈ {0.25, 0.50, 0.75, 1.00}; three parametric families; K ∈ {2, 3}. Newton–Raphson convergence: ‖U_n_‖ < 10⁻⁸ (max 500 iterations).
Data source location	National University of Uzbekistan named after Mirzo Ulugbek, University str. 4, Tashkent 100,174, Uzbekistan.
Data accessibility	Repository name: Mendeley Data Data identification number: 10.17632/mv42gshrcv.2 Direct URL: https://data.mendeley.com/datasets/mv42gshrcv/2
Related research article	[1] Nurmukhamedova N.S. Asymptotic theory of statistical inference in parametric competing risks models under hybrid censoring. Doctoral dissertation (DSc), National University of Uzbekistan, 2026. Preprint: https://zenodo.org/doi/10.5281/zenodo.20792457.

## 1 Value of the Data

• Why are these data useful? Generating these simulation results from scratch requires approximately 4 hours of CPU time on an 8-core machine (or ≈32 hours single-core). The deposited CSV files give researchers immediate access to 64 (n, β, model) experimental settings — covering exponential, Weibull (α = 2), and Gompertz families with K ∈ {2, 3} competing risks — without rerunning computations. Empirical values (bias, RMSE, Type I error, power) are paired with theoretical predictions from $I\left(\theta_{0}\right)=\beta I^{M}\left(\theta_{0}\right)$ [1] so that the finite-sample gap relative to the asymptotic approximation is directly readable from the files.
• Who can benefit from these data? (a) Reliability engineering: practitioners designing accelerated life tests under hybrid censoring can read minimum sample size n* from table_sample_size.csv without additional simulation. (b) Biostatistics and clinical trials: researchers planning studies with competing causes of failure can use the power tables to set sample sizes under realistic censoring fractions. (c) Theoretical statisticians: the K = 3 scenario and the Clayton copula robustness table serve as benchmarks for new estimators or test statistics in multi-cause survival models [2,3].
• How can these data be used for further insights? (a) Benchmarking: new test statistics for competing risks under hybrid censoring can be compared against the Wald, LR, and score tables without rebuilding the baseline simulation. (b) Calibration: table_ci_coverage.csv quantifies at which (n, β) the asymptotic normal approximation is adequate, guiding the choice between asymptotic and bootstrap inference. (c) Reproducibility: diagnostics.csv records non-convergent replications per setting, enabling direct comparison with alternative implementations.
• The dataset includes the K = 3 competing-risks scenario (θ₀ = (1.0, 0.5, 0.3), β = 0.75) in table_k3_rmse.csv. Published simulation studies for three simultaneous causes under hybrid censoring are sparse; this file provides a numerical reference for that configuration [4,5].
• All generation scripts (simulate_crm.py, bootstrap_ci.py, lung_analysis.R) and the full experimental design are included so that the dataset can be extended to new (*n*, β, model) settings or alternative distributions.

## 2 Background

Hybrid censoring combines a pre-specified time limit T₀ (Type I) and a pre-specified failure count r (Type II): observation stops at $\tau_{n}=\text{min}\left(X_{\left(r\right)},T_{0}\right)$. This scheme is common in accelerated life testing and clinical trials [4,6]. In parametric competing risks models [2,3,7], each subject can fail from one of K mutually exclusive causes, each with its own cause-specific hazard $\lambda_{j}\left(t;\theta_{j}\right)$. The doctoral dissertation [1] establishes the local asymptotic normality (LAN) of the log-likelihood process, derives the Fisher information identity $I\left(\theta_{0}\right)=\beta I^{M}\left(\theta_{0}\right)$ where β is the failure fraction [8,9], and proves asymptotic equivalence of the Wald, likelihood-ratio, and score statistics. Complementary results on Fisher information in incomplete observations are discussed in [10]. Related estimation work under random censoring in competing risks models was presented in [11], maximum likelihood and Bayesian estimation under right-censoring was compared in [12], and parameter estimation under hybrid censoring was studied in [5]. A preprint is available at https://zenodo.org/doi/10.5281/zenodo.20792457. The present dataset was generated to provide numerical values corresponding to these results and to extend the grid to K = 3 risks, Clayton copula dependence, and bootstrap confidence intervals.

The novel contributions of this dataset are: (i) a comprehensive Monte Carlo simulation study for parametric competing risks under hybrid censoring, covering three parametric families and K ∈ {2, 3}; (ii) empirical validation of the Fisher information identity $I\left(\theta_{0}\right)=\beta I^{M}\left(\theta_{0}\right)$ [1] across 64 experimental settings; (iii) power and size tables for the Wald, LR, and score tests under a fixed alternative with non-centrality λ* [8,9]; (iv) parametric bootstrap CI coverage results; (v) robustness checks under Clayton copula dependence; and (vi) a fully reproducible workflow with all code and data publicly available in Mendeley Data (DOI: 10.17632/mv42gshrcv.2).

The dataset documentation is organised as follows. The Data Description section details the file inventory and column schemas. The Experimental Design section describes the simulation methodology, parametric models, and statistical procedures (Sections 3.1–3.10). The Limitations section identifies conditions under which the asymptotic approximations may be less accurate and directions for future extension.

## 3 Data Description

***Repository structure***

The archive CRM_MendeleyData_v2.zip contains:

• README.md — variable definitions, column descriptions, software requirements, runtime estimates
• data/ — 13 CSV files described in Table A

**Table A:** File inventory. Structural description of all files. Full data in Mendeley Data (10.17632/mv42gshrcv.2); sample rows in Table B.

File name	Format	Content description
*table_bias_rmse.csv*	.csv	Bias and RMSE of MLE. Columns: model, K, n, β, param, true_value, mean_estimate, bias, rmse_emp, rmse_theor
*table_power_wald.csv*	.csv	Power of W_n_ under fixed alternative. Columns: model, n, β, power_emp, power_theor, lambda_star, alternative
*table_size_all_tests.csv*	.csv	Type I error of W_n_, LR_n_, S_n_. Columns: model, n, β, size_wald, size_lr, size_score
*table_power_three_tests.csv*	.csv	Simultaneous size and power of all three statistics under fixed alternative. Columns: model, n, beta, h_fixed, size_lr, size_wald, size_score, power_lr, power_wald, power_score, lambda_star, pi_theor
*table_fisher_info.csv*	.csv	Estimated vs. theoretical Fisher information. Columns: model, n, beta, Ihat_11, Itheor_11, rel_error_pct, rmse_Ihat
*table_ci_coverage.csv*	.csv	95 % CI coverage and length. Columns: model, n, beta, method, coverage, mean_length, R_boot, R_sim
*table_k3_rmse.csv*	.csv	RMSE for K = 3 risks. Columns: model, K, n, beta, rmse_theta1_emp, rmse_theta1_theor, rmse_theta2_emp, rmse_theta2_theor, rmse_theta3_emp, rmse_theta3_theor
*table_copula_robustness.csv*	.csv	Type I error under Clayton copula. Columns: theta_cop, dependence_type, size_lr, size_wald, bias_theta1, n, model
*table_hybrid_vs_typeII.csv*	.csv	Type I error: hybrid vs. pure Type II. Columns: r_over_n, n, T0_typeII, T0_hybrid, model, size_lr_typeII, size_wald_typeII, size_score_typeII, size_lr_hybrid, size_wald_hybrid, size_score_hybrid
*table_sample_size.csv*	.csv	Required n* for precision δ = 0.1. Columns: p, T0, beta, n_star_theta1, n_star_theta2, model, delta, formula
*lung_params.csv*	.csv	MLE and 95 % CI for survival::lung (n = 228, K = 2). Columns: model, param, estimate, ci_lower, ci_upper, log_lik, AIC
*lung_cif.csv*	.csv	CIF at t ∈ {180, 365, 548, 730} days. Columns: t, CIF_CRM_exp, CIF_CRM_weib, CIF_AJ, CIF_KM
*diagnostics.csv*	.csv	Per-setting count of non-convergent Newton–Raphson runs. Columns: model, n, β, K, n_nonconv, pct_nonconv
*simulate_crm.py*	.py	Main simulation script. Usage: python simulate_crm.py –seed 42 –R 5000 –n_jobs −1
*bootstrap_ci.py*	.py	Parametric bootstrap CI. Usage: python bootstrap_ci.py –R_boot 999
*lung_analysis.R*	.R	Lung analysis: exponential and Weibull CRM; Aalen–Johansen CIF comparison

**Table B:** Sample rows from table_power_wald.csv. Four representative rows. All from the exponential model. The non-centrality $\lambda_{n}^{*}=n\delta^{2}\beta {\left[I^{M}\left(\theta_{0}\right)\right]}_{11}$ [1,8] grows with n under the fixed alternative δ = 0.278.

model	n	β	power_emp	power_theor	λ*	alternative
exponential	50	0.50	0.207	0.206	1.2869	fixed, δ=0.278
exponential	100	0.75	0.493	0.502	3.8607	fixed, δ=0.278
exponential	200	0.75	0.794	0.794	7.7214	fixed, δ=0.278
exponential	500	0.75	0.992	0.993	19.3035	fixed, δ=0.278
• code/ — three executable scripts (simulate_crm.py, bootstrap_ci.py, lung_analysis.R)

## 4 Experimental Design, Materials and Methods

### 4.1 Competing risks model and hybrid censoring

For i=1,…,n, failure times $X_{i}\;$from overall survival function [2,7]

(1) $$\bar{F}\left(t;\theta_{0}\right)\;=\;\text{exp}\;\left(-\int_{0}^{t}\sum_{j=1}^{K}\lambda_{j}\left(s;\theta_{j}\right)ds\right)$$

and cause $\delta_{i}\in \left\{1,...,K\right\}$ with $P\left(\delta_{i}=j|X_{i}=t\right)=\lambda_{j}\left(t\right)/\Sigma_{1}\lambda_{1}\left(t\right)$.

Hybrid stopping time [4,6]:

(2) $$\tau_{n}=\text{min}\;\left(X_{\left(r\right)},\;T_{0}\right),\;r\;=\left\lfloor \beta n\right\rfloor$$

Observable data:

(3) $$Z_{i}\;=\;X_{i}\;\wedge \;\tau_{n},\;\Delta_{i}^{\left(j\right)}\;=\;1\left\{X_{i}\;\leq \;\tau_{n},\;\delta_{i}\;=\;j\right\}$$

### 4.2 Parametric families

**Exponential:** The exponential model has constant hazard with θ₁₀ = 1.0, θ₂₀ = 0.5 for K = 2 and θ₃₀ = 0.3 for K = 3 [7]:

(4) $$\lambda_{j}\left(t;\theta_{j}\right)\;=\;\theta_{j}$$

**Weibull:** The Weibull model has hazard [2]

(5) $$\lambda_{j}\left(t;\alpha_{j},\;\theta_{j}\right)\;=\;\alpha_{j}\theta_{j}t^{\alpha_{j}\;-\;1}$$

with shape fixed at α = 2 and scales θ₁₀ = 1.0, θ₂₀ = 0.5.

**Gompertz:** The Gompertz model has hazard [7]

(6) $$\lambda_{j}\left(t;\alpha_{j},\;\gamma_{j}\right)=\alpha_{j}\exp\left(\gamma_{j}t\right)$$

with α₁₀ = 0.01, γ₁₀ = 0.5, α₂₀ = 0.01, γ₂₀ = 0.3.

### 4.3 MLE and convergence

MLE ${\hat{\theta }}_{n}$ from Newton–Raphson on U_n_(τ_n_; θ) = 0 [8,9] with method-of-moments starts [1]:

(7) $$\theta_{j}^{\left(0\right)}\;=\;\frac{D_{n}^{\left(j\right)}}{\;\sum {\;}_{i\;=\;1}^{n}Z_{i}}$$

Convergence criterion [1]:

(8) $$∥\;U_{n}\left(\tau_{n};\theta^{\left(k\right)}\right)\;∥\;<\;10^{\;-\;8}$$

Newton–Raphson was preferred over EM-type algorithms [13] because the score and observed information admit closed-form expressions for all three families. Non-convergent replications were replaced with new draws; counts are documented in diagnostics.csv; the maximum non-convergence counts per model group are summarised in Table C.

**Table C:** Maximum Newton–Raphson non-convergence count**s**. (out of R = 5000; K = 2). Full details in diagnostics.csv.

Model	n=50, β=0.25	n=50, β=0.50	n=100, β=0.25	n≥100, β≥0.50
Exponential	7 (0.14%)	1 (0.02%)	0 (0.00%)	0 (0.00%)
Weibull	**12 (0.24%)**	2 (0.04%)	2 (0.04%)	0 (0.00%)
Gompertz	9 (0.18%)	1 (0.02%)	1 (0.02%)	0 (0.00%)

### 4.4 Test statistics

Hypothesis: H₀: θ₁ = θ₁₀ vs. a fixed alternative H₁: θ₁ = θ₁₀ + δ with shift δ = 0.278 [8,9]. Under H₁ all three statistics are asymptotically non-central $\chi_{1}^{2}$ with non-centrality $\lambda_{n}^{*}$ that grows linearly in *n* (Eq. (13)):

**Wald statistic** [9]**:**

(9) $$W_{n}\;=\;n{\left(R{\hat{\theta }}_{n}\;-\;r\right)}^{\;⊤\;}{\left[R{\hat{I}}_{n}^{\;-\;1}R^{\;⊤\;}\right]}^{\;-\;1}\left(R{\hat{\theta }}_{n}\;-\;r\right)$$

**Likelihood-ratio statistic** [9]**:**

(10) $$LR_{n}\;=\;2\left[\ell_{n}\left(\tau_{n};{\hat{\theta }}_{n}\right)\;-\;\ell_{n}\left(\tau_{n};{\tilde{\theta }}_{n}\right)\right]$$

**Score (Rao) statistic** [8]**:**

(11) $$S_{n}=n^{-1}U_{n}{\left(\tau_{n};{\tilde{\theta }}_{n}\right)}^{⊤}{\hat{I}}_{n}^{-1}U_{n}\left(\tau_{n};{\tilde{\theta }}_{n}\right)$$

where θ̃_n_ is the restricted MLE under H₀. The non-centrality parameter is [1,8]:

(12) $$\lambda_{n}^{*}=n\delta^{2}\beta {\left[I_{M}\left(\theta_{0}\right)\right]}_{11}$$

Theoretical power [8,9]:

(13) $$\pi^{*}=1-F_{\chi_{1}^{2}\left(\lambda_{n}^{*}\right)}\left(\chi_{1,\;0.95}^{2}\right)$$

### 4.5 Bootstrap procedures

**Parametric bootstrap CI.** For each of Rᵇ = 999 bootstrap samples a dataset is drawn from $P_{{\hat{\theta }}_{n}}$. The CI is the empirical percentile of $\left({\hat{\theta }}_{1}^{*}-{\hat{\theta }}_{1}\right)$.

Wild (martingale) bootstrap [14]. Rademacher weights ${\varepsilon_{i}}^{\left(b\right)}\;\in \;\left\{-1,\;+1\right\}$ multiplied by estimated martingale increments $d{\hat{M}}_{ji}=dN_{ji}-Y_{i}\lambda_{j}\;ds$:

(14) $$U^{\left(b\right)}\left(\tau_{n};\theta \right)=\;\sum_{i\;=\;1}^{n}\sum_{j\;=\;1}^{K}\varepsilon_{i}^{\left(b\right)}\int_{0}^{\tau_{n}}\ell_{j}\left(s;{\hat{\theta }}_{jn}\right)d{\hat{M}}_{ji}\left(s;{\hat{\theta }}_{jn}\right)$$

### 4.6 Parallel random number generation

numpy.random.SeedSequence [15] with base seed 42. Worker k uses default_rng(SeedSequence(42).spawn(P)[k]). joblib.Parallel (backend = “locky”). Guarantees independent streams and exact reproducibility regardless of CPU count.

### 4.7 Clayton copula generation [7]

(15) $$C_{\theta_{cop}}\left(u,\;v\right)={\left(u^{-\theta_{cop}}+v^{-\theta_{cop}}-1\right)}^{-1/\theta_{cop}}$$

• Step 1. V ∼ Gamma(1/$\theta_{cop}$, 1); U₁, U₂ ∼ Uniform(0, 1).
• Step 2. $W_{k}={\left(-log\;U_{k}/V\right)}^{-1/\theta_{\text{cop}}}$.
• Step 3. $X_{k}=-log\left(1-W_{k}\right)/\theta_{k}$ (exponential marginals).
• Step 4. Apply hybrid censoring: $\tau_{n}=min\left(X_{\left(r\right)},T_{0}\right)$.

$\theta_{\text{cop}}=0$: independent branch ($W_{k}=U_{k}$) to avoid numerical issues.

### 4.8 Real data: survival::lung

The lung dataset [16] (n = 228, 165 deaths, K = 2) from the R survival package [17] was analysed. Hybrid censoring was emulated post-hoc with T₀ = 700 days, r = 150, yielding $\tau_{n}$ = 621 days and β̂_n_ = 0.724.

Note: survival::lung records only death/censored status (status ∈ {1, 2}) and does not contain the specific clinical cause of death. For the K = 2 competing risks illustration, we used sex as a proxy variable (male → risk 1, female → risk 2). This is a methodological demonstration only and does not represent clinically validated cause-specific mortality analysis. A real application would require a dataset with verified cause-of-death records (e.g., a national death register or an adjudicated clinical trial endpoint). This retrospective application of hybrid censoring does not emulate a prospective hybrid-censoring clinical trial design.

In the presence of competing risks the Kaplan–Meier estimator overestimates the cause-specific CIF [3]. The correct nonparametric reference is the Aalen–Johansen estimator [18]:

(16) $$F_{j}\left(t\right)=\int_{0}^{t}S\left(s^{\;-\;}\right)\lambda_{j}\left(s\right)ds$$

### 4.9 Minimum sample size computation

Precision target δ = 0.1 on $\theta_{1}$; smallest n satisfying [1]:

(17) $$RMSE_{theor}\left({\hat{\theta }}_{j};n,\;\beta \right)=\frac{{\left(\beta {\left[I_{M}\left(\theta_{0}\right)\right]}_{jj}\right)}^{-1/2}}{\sqrt{n}}\leq \;\delta$$

Closed-form for exponential model [1]:

(18) $$n^{\;*\;}\;=\;\left[\frac{\theta_{10}\left(\theta_{10}\;+\;\theta_{20}\right)}{\delta^{2}\beta }\right]$$

For Weibull and Gompertz, $n^{\;*\;}$ obtained by grid search over n ∈ {10, …, 2000}. The columns p and T0 in table_sample_size.csv record the target failure fraction r/n and the hybrid censoring time limit, respectively; beta records β = F(τ*).

### 4.10 Software and runtime

Python 3.11 (NumPy 1.24, SciPy 1.11, joblib 1.3, matplotlib 3.8). R 4.3.2 (survival 3.5–3, cmprsk 2.2–11 [19]). Hardware: AMD Ryzen 9 5900X, 32 GB RAM, Ubuntu 22.04. Estimated runtime on 8 cores (joblib.Parallel, –n_jobs −1): ≈4 hours for simulate_crm.py (all main simulation settings) and an additional ≈2 hours for bootstrap_ci.py. The simulation is parallelised using joblib.Parallel (–n_jobs −1; all available cores). For users with limited hardware, sequential execution (–n_jobs 1) requires approximately 32 hours for the main simulation and ≈16 hours for the bootstrap CI table (≈48 hours total on a single core). A minimal working example is provided in README.md.

## Limitations

**Selection bias due to convergence conditioning.** Non-convergent Newton–Raphson replications were replaced with new draws so that exactly R = 5000 results are recorded per setting. At n ≥ 100 and β ≥ 0.50, pct_nonconv is below 0.1 % and the conditioning effect is negligible. At small n and low β the procedure may modestly understate RMSE and Type I error. Users should consult diagnostics.csv before drawing conclusions from settings with pct_nonconv > 1 %.

**Weibull shape restriction.** The Weibull simulations cover only shape parameter α = 2. The case α < 1 requires a separate regularity argument and is outside the scope of this dataset.

**Small-sample and high-censoring limitations.** At n < 50 and β ≤ 0.25 the asymptotic regime has not been reached: Type I error exceeds 0.07 and bootstrap CI coverage falls below 0.93 at n = 30 (see table_size_all_tests.csv and table_ci_coverage.csv). In that regime bootstrap quantiles should be used rather than asymptotic normal quantiles.

**Copula and marginal family coverage.** The Clayton copula robustness analysis covers only exponential marginals; Weibull and Gompertz marginals under copula dependence were not included because frailty-copula generation requires numerical CDF inversion, increasing per-setting runtime by a factor of ≈15. The Gumbel and Frank copulas are also not included.

**Lung dataset application.** The survival::lung application uses sex as a proxy for cause of death; this is a methodological demonstration only and should not be interpreted as clinically valid inference. A real competing risks analysis would require a dataset with verified cause-of-death records.

**Scope of theoretical predictions.** Theoretical predictions are derived from I(θ₀) = β Iᴹ(θ₀) [1]. For the exponential model this can be independently verified: [Iᴹ]₁₁ = 1/(θ₁λ₀), so RMSE_theor = θ₁/√(β n).

**Future research directions.** Future work should extend the simulation framework to: (i) Weibull and Gompertz marginals under copula dependence; (ii) adaptive hybrid censoring schemes; (iii) higher-dimensional competing risks (K > 3); and (iv) semi-parametric models such as the Cox proportional hazards model. The dataset presented here can serve as a benchmark for new estimation methods and test statistics in competing risks analysis.

## Ethics Statement

No human subjects, animal experiments, or social media data were involved in the generation of the simulation dataset. The lung cancer dataset used in §3.8 is publicly available in the R survival package [17] and was originally collected with patient consent as described in [16]. The author has read and follows the ethical requirements for publication in Data in Brief.

## Data Availability

Mendeley DataSimulation dataset on power and size of Wald, likelihood-ratio, and score test statistics in parametric competing risks models under hybrid censoring (Original data)
